# Egg White Hydrolysate as a functional food ingredient to prevent cognitive dysfunction in rats following long-term exposure to aluminum

**DOI:** 10.1038/s41598-018-38226-7

**Published:** 2019-02-12

**Authors:** Caroline Silveira Martinez, Caroline D. C. Alterman, Gema Vera, Antonio Márquez, José-A Uranga, Franck Maciel Peçanha, Dalton Valentim Vassallo, Christopher Exley, Pâmela B. Mello-Carpes, Marta Miguel, Giulia Alessandra Wiggers

**Affiliations:** 1 Graduate Program in Biochemistry, Universidade Federal do Pampa, BR 472–Km 592, PO box 118. Zip Code: 97500-970, Uruguaiana, Rio Grande do Sul Brazil; 2 Department of Ciencias Básicas de la Salud, Universidad Rey Juan Carlos, Avda. de Atenas s/n 28922, Alcorcón, Spain; 3 Departments of Physiological Sciences, Universidade Federal do Espírito Santo and School of Medicine of Santa Casa de Misericórdia (EMESCAM), Av. Marechal Campos 1468, Zip Code: 29040-090, Vitória, Espírito Santo Brazil; 4 The Birchall Centre, Lennard-Jones Laboratories, Keele University, Staffordshire, ST5 5BG UK; 5 Bioactivity and Food Analysis Laboratory, Instituto de Investigación en Ciencias de la Alimentación, Nicolás Cabrera, 9, 28049, Campus Universitario de Cantoblanco, Madrid, Spain; 6 Present Address: Equipe MitoLab, Institut MitoVasc, Université d’Angers, CHU Bât IRIS/IBS Rue des Capucins, 49933 Angers cedex 9, France

## Abstract

Aluminum (Al), which is omnipresent in human life, is a potent neurotoxin. Here, we have tested the potential for Egg White Hydrolysate (EWH) to protect against changes in cognitive function in rats exposed to both high and low levels of Al. Indeed, EWH has been previously shown to improve the negative effects induced by chronic exposure to heavy metals. Male *Wistar* rats received orally: Group 1) Low aluminum level (AlCl_3_ at a dose of 8.3 mg/kg b.w. during 60 days) with or without EWH treatment (1 g/kg/day); Group 2) High aluminum level (AlCl_3_ at a dose of 100 mg/kg b.w. during 42 days) with or without EWH treatment (1 g/kg/day). After 60 or 42 days of exposure, rats exposed to Al and EWH did not show memory or cognitive dysfunction as was observed in Al-treated animals. Indeed, co-treatment with EWH prevented catalepsy, hippocampal oxidative stress, cholinergic dysfunction and increased number of activated microglia and COX-2-positive cells induced by Al exposure. Altogether, since hippocampal inflammation and oxidative damage were partially prevented by EWH, our results suggest that it could be used as a protective agent against the detrimental effects of long term exposure to Al.

## Introduction

Aluminum (Al) is omnipresent in modern life without any known biological beneficial effect^1^. The human body burden of Al increases every single day due to numerous anthropogenic and natural sources of Al^2,3^. Therefore, the Provisional Tolerable Weekly Intake^4,5^ of Al for humans (1 mg Al/kg body weight -b.w.) is exceeded for a significant part of the world population^6,7^. The consequences of the enhanced human body burden of Al are not entirely clear^2^, but may have implications for human disease including neurological disorders such as Alzheimer’s disease (AD)^8–10^, cardiovascular disease^11,12^ and reproductive dysfunction^13,14^.

Al is a widespread neurotoxin associated with cognitive and motor impairments, mostly related with neurodegenerative diseases^15,16^. For many years Al has been implicated in the etiology of AD in the so-called “aluminum hypothesis in AD” and now the most recent research has described how it is involved in the onset, progression and aggressive nature of AD^8,10^. However, while a role for Al in AD is now more certain we still do not understand the predominant toxic mechanism. The toxicity of Al has been related to its pro-oxidant activity, acting through the formation of an Al-superoxide radical cation^17^ capable of reducing Fe(III) to Fe(II) inducing the Fenton reaction^18^.

Due to unanswered questions regarding the human body burden of Al and its real consequences, there is an urgent need for prevention and therapy and, preferably without considerable adverse effects such as disrupting essential metals. In this sense, Egg White Hydrolysate (EWH) bioactive peptides, obtained after enzymatic hydrolysis with pepsin^19^, could be beneficial to counteract the negative effects of Al in human disease. Previously, we have demonstrated the ability of EWH to counteract health effects induced by different conditions such as cardiometabolic dysfunction and heavy metal exposure^19–22^. The protective effects of EWH seem to be related to its antioxidant and anti-inflammatory properties^22–24^.

The behavioral effects of Al exposure on experimental rodents have been studied and, at high levels, Al has been used as an animal model of AD^25–27^. Al-exposed rats at 100 mg Al/kg/day, develop progressive deterioration of spatial memory^26,27^ and, at 250 mg/kg object recognition memory and sociability were impaired in Al-treated mice^28^. Social interaction impairment was also shown following injection of Al adjuvants in neonatal mice pups during the early period of postnatal development^29^. Recently, we have demonstrated that Al exposure at a level which might be considered equivalent to normal dietary intake was sufficient to promote cognitive dysfunction, such as memory impairment and that these effects were almost the same when we treated rats at a higher (super-dietary level) dose of Al^30^. Herein, we have investigated if EWH is effective in protecting against cognitive function in rats exposed to both a low and high level of dietary Al.

## Methods

### Preparation of EWH

EWH was prepared by pepsin hydrolysis of crude egg white, as previously described^20^. Briefly, commercial pasteurized egg white was hydrolyzed with BC Pepsin 1:3000 (E.C. 3.4.23.1; from pork stomach, E:S: 2:100 w-w, pH 2.0, 38 °C), purchased from Biocatalysts (Cardiff, United Kingdom), for 8 h. Enzyme inactivation was achieved by increasing the pH to 7.0 with 5 N NaOH. The hydrolysate was centrifuged at 2500 g for 15 min. and the supernatants were frozen and lyophilized. The principal components of EWH after pepsin digestion for 8 h were previously determined by reverse-phase liquid chromatography–mass spectrometry (RP-HPLC-MS/MS), peptides: FRADHPFL, RADHPFL, YAEERYPIL, YRGGLEPINF, ESIINF, RDILNQ, IVF, YQIGL, SALAM, FSL^19,31^.

### Animals

Male *Wistar* rats (90 days-old, 360 ± 11.2 g) were obtained from the Charles River Animal Laboratory, Barcelona, Spain. Animals were housed at standard conditions (constant room temperature, humidity, and 12:12 h light-dark) with water and fed *ad libitum*. All experimental protocol were performed in accordance with the guidelines stated by the Brazilian Societies of Experimental Biology and the European and Spanish legislation on care and use of experimental animals (EU Directive 2010/63/EU for animal experiments; R.D. 53/2013). The experimental protocol was approved by the Ethics Committees on Animal Use at both Universidade Federal do Pampa, Uruguaiana, Rio Grande do Sul, Brazil (Process Number: 028/2014) and Universidad Rey Juan Carlos, Madrid, Spain (Process Number: 39/2012).

Male *Wistar* rats were randomly distributed into two main groups according to their Al exposure and received orally and once a day: Group (1) Low aluminum level - rats were divided into 4 subgroups (N = 8) (1a-d) and received for 60 days: (a) Control - ultrapure water as the drinking water (Milli-Q, Merck Millipore Corporation. © 2012 EMD Millipore, Billerica, MA); (b) AlCl_3_ at a dose of 8.3 mg/kg b.w. in the drinking water, representing human Al exposure by diet^30^; (c) EWH - ultrapure water as the drinking water and EWH at 1 g/kg/day by gavage^32^; (d)EWH + AlCl_3_ at 8.3 mg/kg bw; and Group (2) High aluminum level - rats were divided into 4 subgroups (N = 8/each) (2a-d) and received for 42 days: (a) Control – ultrapure water by oral gavage; (b) AlCl_3_ at 100 mg/kg b.w. for by gavage, representing a high level of human exposure to Al^26^; (c) EWH - ultrapure water and EWH at 1 g/kg/day both by gavage^32^; (d) EWH + AlCl_3_ at 100 mg/kg b.w.

Rat body weights, food and water or Al intakes for groups that have received Al in the drinking water (Group 1) were measured once a week. After 60 (Group 1a-d) or 42 (Group 2a-d) days of treatment the animals were submitted to behavioral tests: 1° day: open field, plus maze and hot plate (control behavioral experiments); 2°–6° day: object recognition tests (short and long-term memory). After the exposure period and tests, rats were euthanized and the bilateral hippocampus was quickly dissected out and, one side was processed for histological and/or immunohistochemical studies and the other side was quickly homogenized in 50 mM Tris HCl, pH 7.4, (1/10, w/v), centrifuged (2400 g, 10 min, 4 °C) and frozen at −80 °C for further biochemical determinations.

AlCl_3_.6 H_2_O solution was prepared in ultrapure water being the concentration of each stock solution of 0.034 M (Group 1; 8.3 mg/kg/b.w.) and 0.331 M (Group 2; 100 mg/kg/b.w.). All reagents and salts were obtained from Sigma-Aldrich and Merck (Darmstadt, Germany).

### Control behavior tests

Control behavior tests were performed to guarantee that the procedures adopted in the experiment’s design did not alter rats’ locomotion and exploration, anxiety state and pain threshold, since these alterations could interfere on memory testing. After testing, the results were compared between groups. To evaluate locomotion and exploration the Open Field test was used; the number of crossing and rearings were monitored by 5 min as described previously^33^. The Elevated Plus Maze test was used to evaluate the anxiety behavior; each rat was placed in the maze for 5 min and the time in the open arms of the maze was monitored for each rat as previously described^34^. The hot plate test was used to evaluate the pain threshold; the animals’ time to react was measured as previously described^35^, considering a ceiling of 45 s, to avoid lesions.

### Short and long-term object recognition memory

Object recognition memory test (OR) was used to evaluate the short (STM) and the long-term memory (LTM) of the animals. After the treatments were ended, the OR protocol was started. It was performed in an apparatus (50 cm × 50 cm × 50 cm area built with plastic, plywood and transparent acrylic, as previously described^36^). Initially, the animals were habituated to the arena, being placed in it for 20 min per day during 4 consecutive days. In the fifth day, the OR training was performed; for this, two different objects (a and b) were placed in the apparatus and the rat had 5 minutes to free exploration. The objects were made of different materials, and the exploration was monitored considering sniffing or touching the objects with the nose and/or forepaws by a video camera. STM test was performed 3 h after training, when one of the objects was changed by a new one (c). LTM test was performed 24 h after training, when one of the objects was changed by a new one (d). In each test session, the rat had 5 minutes to freely exploration of the objects. Between each animal’s training or testing session, the arena and the objects were cleaned with 70% ethanol to avoid influences of olfactory stimuli in the results. In each OR session the exploration time of each one of the objects was recorded for further analysis.

### Catalepsy

Catalepsy was measured using a modification of the “ring test”^37^. Rats were hung by their front paws from a rubber-coated metal ring fixed horizontally at a height that allowed their hind paws to just touch the bench. The time taken for the rat to move-off the ring was measured with a cut-off limit of 30 seconds.

### Reactive oxygen species levels

Levels of reactive oxygen species in the hippocampus were determined by the spectrofluorometric method described by Loetchutinat *et al*.^38^ with previous modifications^30^. The ROS levels were expressed as fluorescence units.

### Lipid peroxidation

Lipid peroxidation was measured in the hippocampal tissue as malondialdehyde (MDA) using a colorimetric method, as previously described by Ohkawa *et al*.^39^, with modifications^30^. The results were expressed as nanomoles of MDA per mg of protein.

### Ferric Reducing/Antioxidant Power (FRAP) Assay

The total antioxidant capacity in the hippocampal tissue was measured by FRAP assay^40^, with modifications^30^. Results are presented with particular reference to Trolox equivalents.

### Acetylcholinesterase (AChE) activity

The AChE activity in hippocampal tissue was assessed by the Ellman method^41^, with modifications^30^. Results were expressed as micromoles of acetylthiocholine iodide hydrolyzed/min/mg of protein. Proteins were measured according to Bradford^42^ using bovine serum albumin as a standard.

### Histology analysis

To carry out the histological studies hippocampal tissue was fixed in 10% formaldehyde for 1–2 days. After several intensive washings, tissues were embedded in paraffin, sectioned at 5 µm and stained with hematoxylin/eosin. For the histological study, 10 randomly chosen regions of each hippocampus were analysed as a blinded evaluation, and, studied under a Zeiss Axioskop 2 microscope (Zeiss, Jena, Germany) equipped with the image analysis software package AxioVision 4.6. The analysis was made under the 10X objective.

### Immunohistochemistry

Hippocampus immunohistochemistry was performed on paraffin-embedded sections of 5 µm thickness. De-paraffined slides were washed with phosphate buffered saline (PBS) with 0.05% Tween 20 (Calbiochem, Darmstadt, Germany). Thereafter sections were incubated for 10 min in 3% (*v/v*) hydrogen peroxide to inhibit endogenous peroxidase activity and blocked with fetal bovine serum for 30 minutes to minimize nonspecific binding of the primary antibody. Sections were then incubated overnight at 4 °C with a rabbit polyclonal antibody against microglia (anti-Iba 1, 1:500, Wako Chemicals, USA, Inc) to quantify the number of activated microglia, which is consistent with the presence of inflammation. Sections were also incubated with the primary rabbit polyclonal antibody against cyclooxygenase- 2 (COX-2, 1:75, Santa Cruz Biotechnology, Inc.). After incubation, samples were washed with PBS-Tween. The peroxidase-based kit Masvision (Master Diagnostica, Granada, Spain) was used as secondary antibody. Samples were counterstained with hematoxylin and coverslips were mounted with Eukitt (O. Kindler GmbH & Co, Freiburg, Germany). To determine the level of non-specific staining the preparations were incubated without the primary antibody. COX-2 immunohistochemistry was evaluated for intensity of immunoreactivity on a 0 to 4+ scale. The overall intensity of the staining reaction was scored with 0 indicating no immunoreactivity and no positive cells, 1+ weak immunoreactivity and <10% of positive cells, 2+ mild immunoreactivity and 10–30% of positive cells, 3+ moderate immunoreactivity and 31–60% of positive cells, and 4+ strong immunoreactivity and 61–100% of positive cells, as described by Kohno *et al*.^43^.

### Lumogallion staining for presence of aluminum

Lumogallion staining was performed on formalin-fixed hippocampal tissue using a recently validated method to identify the presence of Al in tissues^44^^,^^45^. Briefly, re-hydrated tissues sections were immediately placed into either 1 mM lumogallion (TCI Europe N.V. Belgium) buffered in 50 mM PIPES, pH 7.4 or the PIPES-buffer alone for auto-fluorescence analyses for 45 minutes. Slides were carefully washed 6 times with PIPES-buffer and rinsed in ultrapure water for 30 seconds. Sections were then incubated with DAPI to identify the cell nucleus (4′,6-Diamidino-2-phenylindole dihydrochloride - DAPI, 1:10.000, Santa Cruz Biotechnology, Inc., Santa Cruz, CA, USA). Afterwards, slides were mounted using an aqueous mounting medium and stored horizontally at 4 °C overnight prior to imaging. Sections of tissues were imaged using a Zeiss Axioskop 2 microscope (Zeiss, Jena, Germany).

### Statistical analysis

Results are expressed as mean ± SD. The time spent exploring each of the objects in the OR test was converted to a percentage of total exploration time; the analysis were made using a one-sample t-test considering a theoretical mean of 50%. The other results were analyzed using one or two way ANOVA followed by Bonferroni *post hoc* test when necessary. Values of P < 0.05 were considered significant.

## Results

### Body weight, fluid and feed intakes

The liquid, feed intakes and body weights of rats were not different between groups at the beginning or end of the treatments (body weight varying from 284.4 ± 12.5 to 468.1 ± 10.5 g - Table 1).

**Table 1:** Body weights and dietary intakes in each of the treatment groups.

Body weight Feed/fluid intakes	Al 8.3 mg/kg + EWH				Al 100 mg/kg + EWH			
	Control	EWH	AlCl_3_	AlCl_3_ + EWH	Control	EWH	AlCl_3_	AlCl_3_ + EWH
Initial body weight (g)	365.6 ± 20.4	385.6 ± 29.7	409.7 ± 28.4	397.7 ± 28.6	284.4 ± 29.3	297.1 ± 25.7	283.1 ± 29.6	309.5 ± 28.4
Final body weight (g)	434.1 ± 25.1	467.6 ± 29.1	468.1 ± 30.2	448.4 ± 22.9	386.9 ± 30.2	413.1 ± 30.6	408.3 ± 29.4	408.5 ± 25.5
Feed intakes (g)	21.4 ± 0.6	22.2 ± 1.5	22.5 ± 0.6	22.2 ± 1.3	20.7 ± 0.8	21.9 ± 1.6	21.4 ± 0.7	21.5 ± 0.5
Fluid intakes (mL)	36.1 ± 2.3	37.7 ± 2.5	36.1 ± 1.6	36.7 ± 0.9	31.4 ± 2.4	32.4 ± 2.8	31.7 ± 1.1	30.6 ± 0.8

Mean and SD are given for n = 8 rats. P > 0.05 (ANOVA).

### Control behavioral testing, STM and LTM results

For group 1 or group 2, Al exposure, the EWH treatment alone or co-treated with Al had no effect in the quantity of crossings and rearings during the open field test (Table 2 – Open field). In the same manner, neither of the treatments affected the permanency of the animals in the open arms during the plus maze test (Table 2 – Plus maze) or in latency time to reaction during the hot plate section (Table 2 – Hot plate).

**Table 2:** Results of control behavioral testing performed by Open Field, Elevated Plus Maze and Hot Plate testing.

Behavioral tasks		Al 8.3 mg/kg + EWH					Al 100 mg/kg + EWH				
		Control	EWH	AlCl3	AlCl_3_ + EWH	F(df); P value	Control	EWH	AlCl3	AlCl_3_ + EWH	F(df); P value
Open Field	Crossings (n)	44.33 ± 28.60	37.70 ± 13.31	41.60 ± 22.75	42.70 ± 15.03	F_(3,35)_ = 0.82 P = 0.48	41.44 ± 19.93	43.80 ± 21.63	53.10 ± 24.64	38.00 ± 22.90	F_(3,35)_ = 1.36 P = 0.26
	Rearings (n)	17.33 ± 9.97	7.70 ± 3.59	13.50 ± 10.72	14.80 ± 7.36	F_(3,35)_ = 0.68 P = 0.56	11.11 ± 5.96	15.60 ± 8.60	14.50 ± 8.84	12.50 ± 5.91	F_(3,35)_ = 2.77 P = 0.09
Plus maze	Time in open arms (s)	15.26 ± 23.06	16.89 ± 10.60	10.43 ± 12.37	19.54 ± 15.60	F_(3,35)_ = 0.27 P = 0.84	20.88 ± 29.73	29.90 ± 13.57	24.08 ± 17.29	26.53 ± 26.06	F_(3,34)_ = 0.47 P = 0.7
Hot plate	Times (s)	13.63 ± 3.33	9.20 ± 4.94	11.50 ± 4.60	12.50 ± 4.35	F_(3,35)_ = 1.09 P = 0.36	12.11 ± 4.64	8.70 ± 4.90	9.40 ± 4.27	9.00 ± 4.34	F_(3,34)_ = 0.43 P = 0.18

Mean and SD are given for n = 8 rats. P > 0.05 (ANOVA).

**Figure 1:**
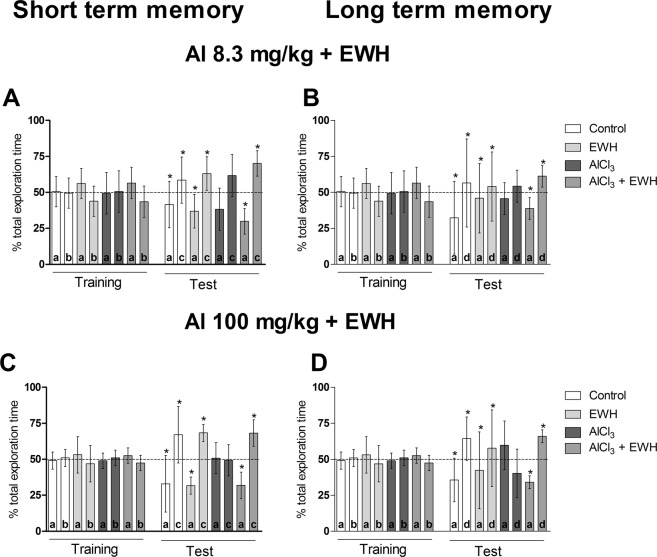
Effects of EWH on short and long-term recognition memory of rats exposure to aluminum. After AlCl_3_ exposure for 60 (8.3 mg/kg bw *per day* – **A**,**B**) or 42 days (100 mg/kg bw *per day* – **C**,**D**), co-treated or not with EWH, object recognition task (OR) was used to evaluate memory. After training in the OR task, the rats’ short (STM) and long-term memory (LTM) are tested, considering test session performed 3 h and 24 h after OR training. During the training session the rats were exposed to two objects (“a” and “b”). In each test session one of the objects were replaced by a new one (“c”, in the STM test, or “d”, in the LTM test). Results are expressed as mean ± SD of the percentage of total exploration time. *P < 0.05 in one-sample t-test; theoretical mean of 50% (n = 8).

During OR training, all rats in both groups spent about 50% of the total exploration time to explore two new objects (a and b) (Group 1: Fig. 1A,B: Cont t_(9)_ = 0.12; P = 0.90, EWH: t_(9)_ = 1.85; P = 0.09; AlCl_3_ (Al) 8.3: t_(8)_ = 0.14; P = 0.88; Al 8.3 + EWH: t_(9)_ = 1.88; P = 0.09; Group 2: 1B, 1 C, 1D: Cont t_(7)_ = 0.44; P = 0.67; EWH: t_(9)_ = 0.77; P = 0.45; Al 100: t_(9)_ = 0.62; P = 0.55; Al 100 + EWH: t_(9)_ = 1.51; P = 0.16), as expected.

However, 3 h after training, in the short-term memory testing sessions, while control (Ct) animals explored the new object more often than the familiar object (Fig. 1A test Cont t_(9)_ = 2.51, P = 0.03; Fig. 1B test Cont t_(7)_ = 2.60, P = 0.03), Al-treated rats (Group 1b and Group 2b) spent a similar time exploring these objects, approximately 50% of the total time (Fig. 1A test Al t_(8)_ = 1.58, P = 0.15; Fig. 1B test t_(9)_ = 0.20, P = 0.84). EWH had no effect per se (Fig. 1A test EWH t_(9)_ = 3.53, P = 0.06; Fig. 1B test t_(9)_ = 9.73, P < 0.0001) while, when rats were treated with Al and EWH (Group 1d and Group 2d), they explored the novel object (c) for a significantly longer period of time (more than 50%Fig. 1A test Al + EWH t_(9)_ = 7.15, P < 0.0009; Fig. 1B test t_(9)_ = 6.25, P = 0.0001), similar to their respective control groups (Group 1a and Group 2a).

We made similar observations in the 24 h test (long - term memory testing sessions). The Al-treated rats (Group 1b and Group 2b) explored both objects for approximately the same amount of time, without differences in the time spent exploring the familiar and the novel object (Fig. 1C test Al t_(9)_ = 1.22, P = 0.25; Fig. 1D test t_(9)_ = 1.82, P = 0.10). When rats were treated with Al and EWH (Group 1d and Group 2d), they spent more than 50% of the total exploration time exploring the new object (c) (Fig. 1C test Al + EWH t_(9)_ = 4.69, P = 0.001; Fig. 1D test t_(9)_ = 11.25, P < 0.0001), similar to their respective control groups (Group 1a and Group 2a). However, 3 h or 24 h after training, in the short and long term memory testing sessions, respectively, the Al-treated rats (Group 1b and Group 2b) spent a similar time exploring the familiar and the novel object (a and c or d) (Fig. 1A–D – test session). While, when rats were treated with Al and EWH (Group 1d and Group 2d), they explored the novel object (c) for more than 50% of the time (Fig. 1A–D – test session), similar to their respective control groups (Group 1a and Group 2a).

**Figure 2:**
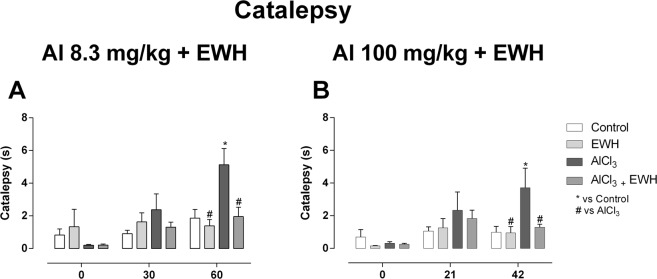
Effect of EWH on catalepsy development in Al-treated rats. The catalepsy analysis was performed before (day 0), in the middle (30 or 21 days) and end of the treatments (60 or 42 days) with AlCl_3_ for 60 (8.3 mg/kg bw *per day* – **A**) or 42 days (100 mg/kg bw *per day* – **B**), co-treated or not with EWH. Results are expressed as mean ± SD, n = 8, *P < 0.05 compared with their corresponding controls, ^#^P* < *0.05 compared with AlCl_3_ group (two-way ANOVA and Bonferroni as post-hoc test).

In the catalepsy test, a main effect for the groups was observed on rats exposed to low levels of Al (Group 1b: F_(3,84)_ = 3.577; P = 0.01; Fig. 2A) or high (Group 2b: F_(3,84)_ = 3.421; P = 0.02; Fig. 2B). Additionally, a main effect of the time was observed on rats exposed to low levels of Al (Group 1b: F_(2,84)_ = 10.19; P = 0.0001, Fig. 2A) or high (Group 2b: F_(2,84)_ = 7.324; P = 0.001; Fig. 2B). Interaction effect was observed on rats exposed to low (Group 1b: F_(6,84)_ = 3.134; P = 0.008; Fig. 2A), but not on rats treat with high levels of Al (Group 2b: F_(6,84)_ = 1.629; P = 0.14; Fig. 2B).

Animals exposed to Al during long-term (Group 1b) showed increased latency time for reaction in the test when compared to their respective control group, being positive for catalepsy (P < 0.01; Fig. 2A). However, rats treated with low levels of Al and co-treated with EWH demonstrated a motor behavior different from Al non-treat animals and similar to the control and EWH rats (P > 0.05 for control vs. ACl_3 + _EWH_;_ P < 0.01 for ACl_3_ vs. AlCl_3_ + EWH; Fig. 2A).

Rats exposed to high levels of Al for 42 days (Group 2b) showed increased latency time for reaction in the test when compared to their respective control group, being positive for catalepsy (P < 0.01; Fig. 2B). However, rats treated with low levels of Al and also co-treated with EWH demonstrated a motor behavior different from Al non-treat animals and similar to the control and EWH rats (P > 0.05 for control vs. ACl_3 + _EWH_;_ P < 0.05 for ACl_3_ vs. AlCl_3_ + EWH; Fig. 2B).

### Oxidative stress and AChE activity

**Figure 3:**
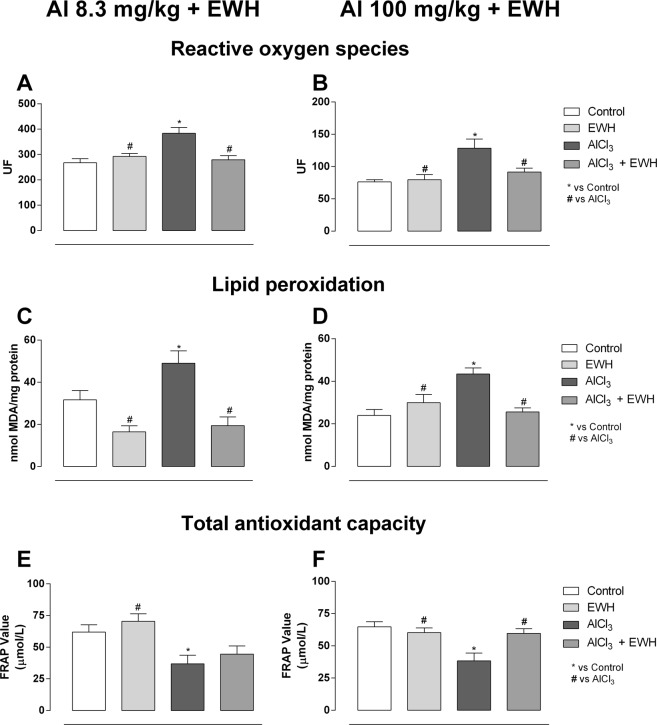
Effect of EWH on oxidative stress assessments in Al-treated rats. Reactive oxygen species (ROS), lipid peroxidation and total antioxidant capacity (FRAP- Ferric Reducing/Antioxidant Power) levels in hippocampus of rats treated with AlCl_3_ for 60 (8.3 mg/kg bw *per day* – **A**,**C**,**E**) or 42 days (100 mg/kg bw *per day* – **B**,**D**,**F**), co-treated or not with EWH. Results are expressed as mean ± SD, n = 8, *P < 0.05 compared with their corresponding controls, ^#^P < 0.05 compared with AlCl_3_ group (two-way ANOVA and Bonferroni as post-hoc test).

Al treatment at low (Group 1b) and high doses (Group 2b) raised the ROS and lipid peroxidation levels and, decreased the hippocampal antioxidant capacity (ROS Fig. 3A: Al 8.3 vs Ct P = 0.0014; Fig. 3B: Al 100 vs Ct P = 0.0026; Lipid peroxidation Fig. 3C: Al 8.3 vs Ct P = 0.0437; Fig. 3D: Al 100 vs Ct P = 0.0013; Total antioxidant capacity Fig. 3E: Al 8.3 vs Ct P = 0.0264; Fig. 3F: Al 100 vs Ct P = 0.0010). Rats exposed to both Al and EWH (Group 1d and Group 2d) showed oxidative stress biomarkers similar to the levels found in the control and EWH rats (ROS Fig. 3A: Al 8.3 + EWH vs Al P = 0.0038; Fig. 3B: Al 100 + EWH vs Al P = 0.0464; Lipid peroxidation Fig. 3C: Al 8.3 + EWH vs Al P = 0.0029; Fig. 3D: Al 100 + EWH vs Al P > 0.9999; Total antioxidant capacity Fig. 3F: Al 100 + EWH vs Al P = 0.0373).

**Figure 4:**
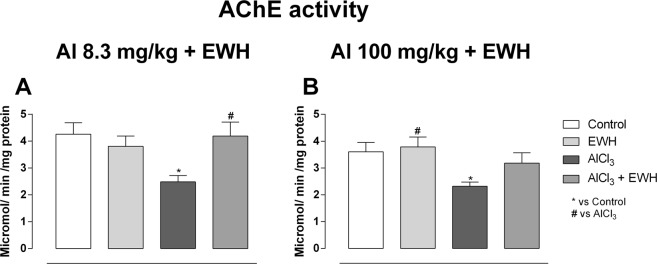
Effect of EWH on acetylcholinesterase (AChE) activity in Al-treated rats. Values of AChE activity on hippocampus of rats treated with AlCl_3_ for 60 (8.3 mg/kg bw *per day* – **A**) or 42 days (100 mg/kg bw *per day* – **B**), co-treated or not with EWH. Results are expressed as mean ± SD, n = 8, *P < 0.05 compared with their corresponding controls (two-way ANOVA and Bonferroni as post-hoc test).

Al-treated rats at low and high doses showed an inhibition of 42% (Group 1b) and 37% (Group 2b) on AChE activity, respectively (Fig. 4A: Al 8.3 vs Ct P = 0.0057; Fig. 4B: Al 100 vs Ct P = 0.0122). Al-exposed rats at 8.3 mg/kg (Group 1a) and co-treated with EWH (Group 1d) showed an inhibition of 1.7% in the enzyme activity and, Al-exposed rats at100 mg/kg (Group 2b) and co-treated with EWH (Group 2d) showed a not statistical difference inhibition of 11.6% on AChE activity (Fig. 4A: Al 8.3 + EWH vs Al P = 0.0292; Fig. 4B; Al 100 + EWH vs Al P = 0.4228).

### Histology and neuroinflammation analysis – microglia and COX-2

**Figure 5:**
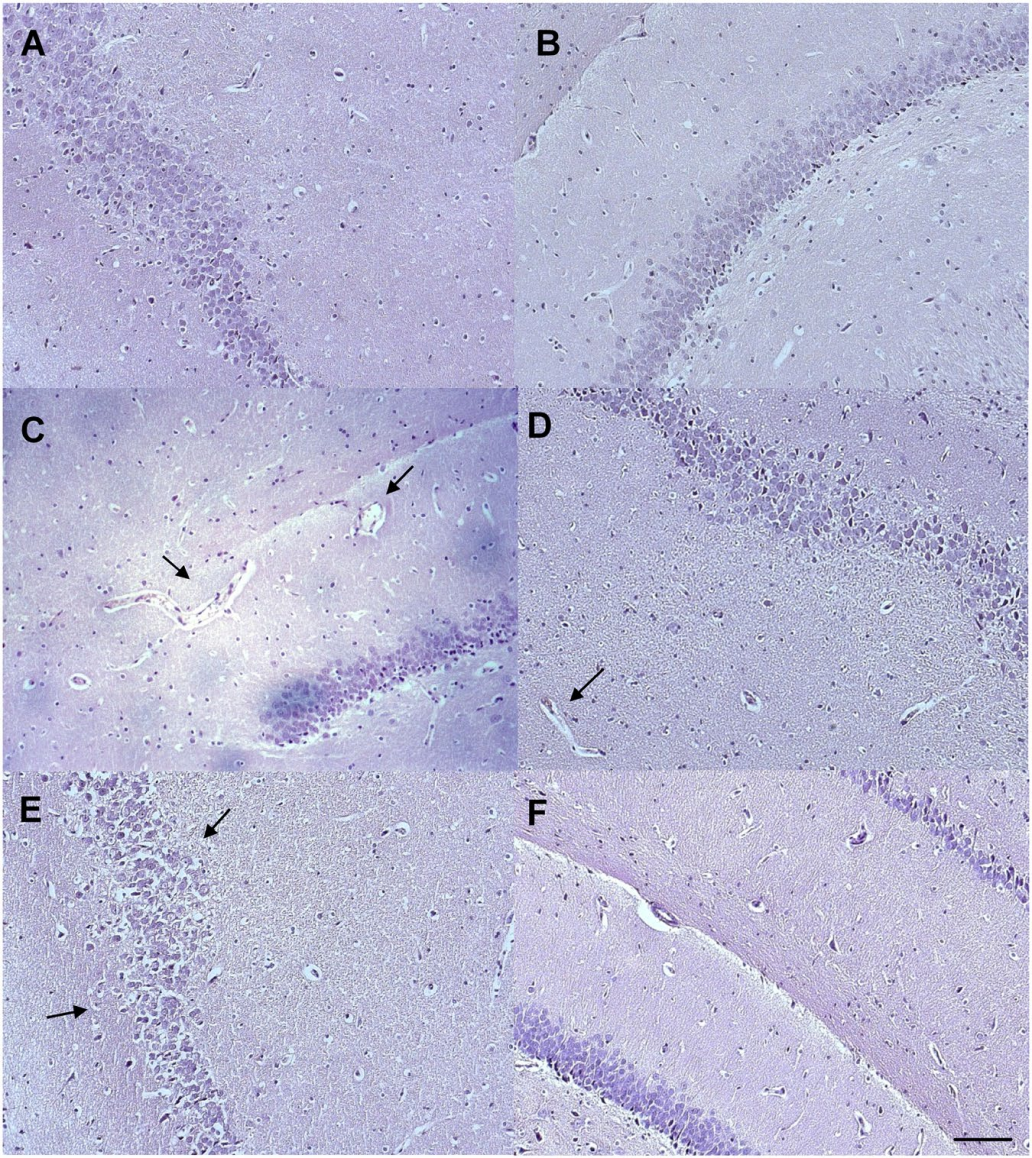
Effect of EWH on hippocampus histopathology in Al-treated rats. Normal histoarchitecture in hippocampus of control (**A**) and EWH (**B**) groups. Tissues sections of Al-treated rats with 8.3 mg/kg b.w. (**C**) and with 100 mg/kg b.w. (**E**) Arrows indicate blood vessels in C and vacuolarized cells in E. The co-treatment with EWH ameliorates the damage after Al exposure with 8.3 mg/kg (**D**) and 100 mg/kg (**F**). Scale bar: 100 µm (objective X10).

Histology showed normal histoarchitecture of the hippocampus in control (Group 1a and Group 2a) and EWH rats (Group 1c and Group 2c) (Fig. 5A,B). On the contrary, Al exposure at an equivalent human dietary level (Group 1b) and at a high level of human exposure (Group 2b) impaired the normal histoarchitecture of the hippocampus, these effects not being restricted to specific sub-regions of the hippocampus, but similar across the whole tissue. Specifically, Al at the lower dose (Group 1a) promoted alterations in vessel morphology and Al at 100 mg/kg (Group 2b) induced granulovacuolar structures in the cellular cytoplasm (Fig. 5C,E, respectively). Rats exposed to Al and EWH (Group 1d and Group 2d) showed histology similar to the control groups (Fig. 5D,F).

**Figure 6:**
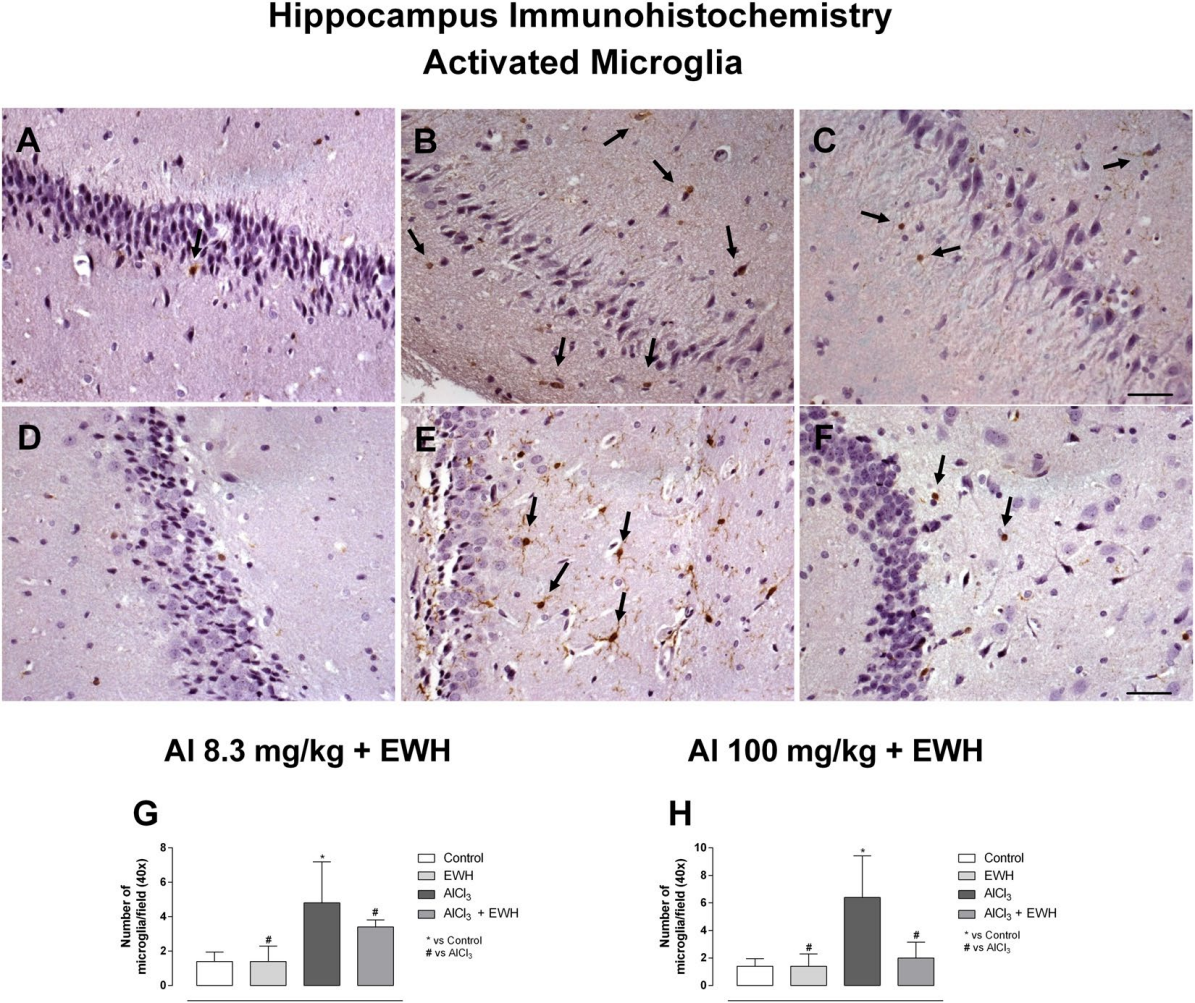
Effect of EWH on hippocampus microglia in Al-treated rats. Activated microglia (arrows) in hippocampus of controls group (**A**,**D**), Al at 8.3 mg/kg b.w. (**B**) and Al at 100 mg/kg b.w. (**E**), EWH + Al 8.3 (**C**) and EWH + Al 100 (**F**) detected with the Iba-1 antibody. Scale bar 50 µm. Average numbers of activate microglia per field (objective X40) of rats treated with AlCl_3_ at 8.3 (**G**) or 100 (**H**), co-treated or not with EWH. Results are expressed as mean ± SD, n = 8, *P < 0.05 compared with their corresponding controls (two-way ANOVA and Bonferroni as post-hoc test).

**Figure 7:**
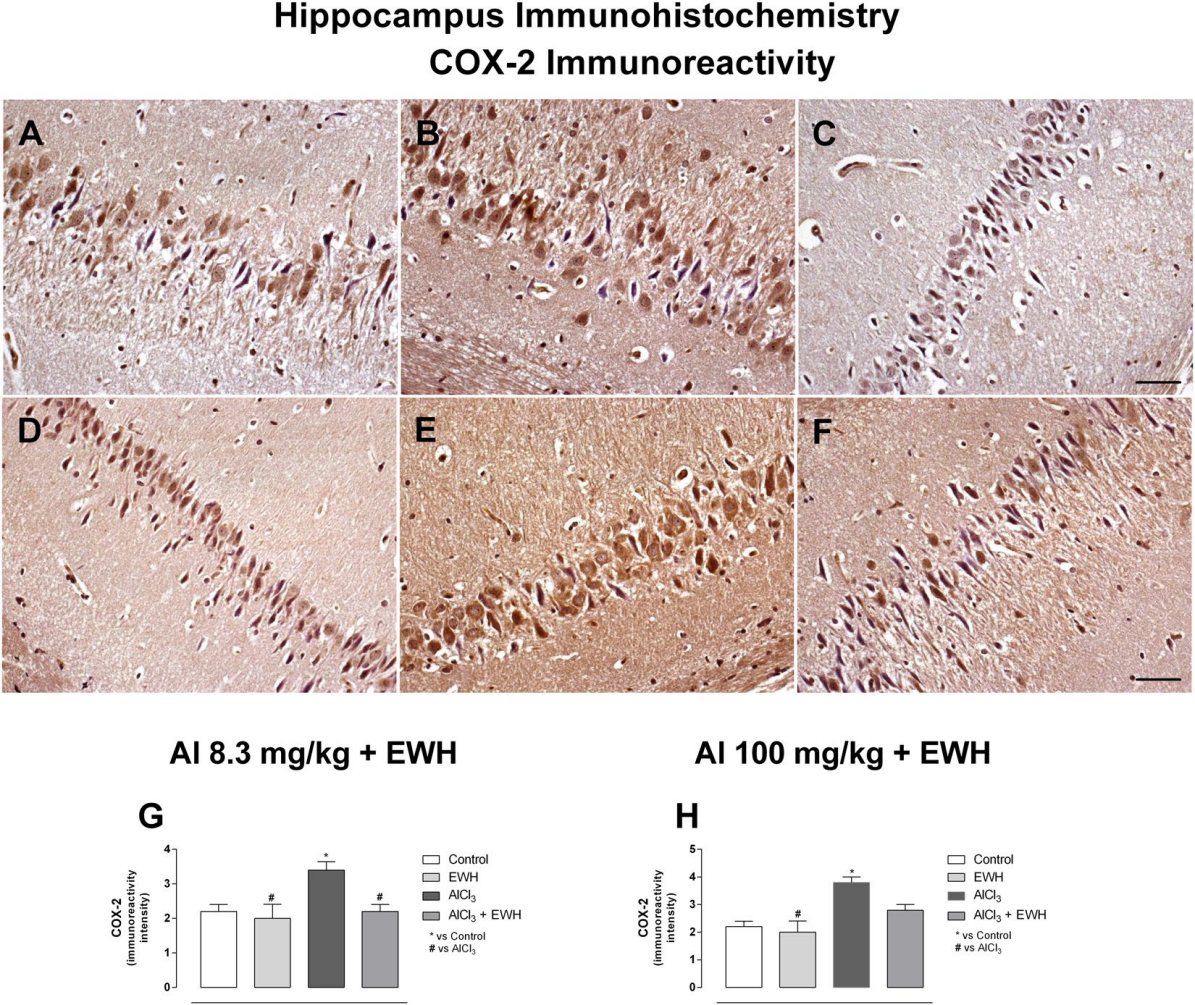
Effect of EWH on hippocampus COX-2 immunoreactivity in Al-treated rats. Immunoreactivity for COX-2 (arrows) in hippocampus of controls group (**A** and **D**), Al at 8.3 mg/kg b.w. (**B**) and Al at 100 mg/kg b.w. (**E**), EWH + Al 8.3 (**C**) and EWH + Al 100 (**F**) detected by immunohistochemistry. Scale bar 50 µm and objective X20. COX-2 score for intensity of immunoreactivity (0 to 4 + scale) (objective X40) of rats treated with AlCl_3_ at 8.3 (**G**) or 100 (**H**), co-treated or not with EWH. Results are expressed as mean ± SD, n = 8, *P < 0.05 compared with their corresponding controls (two-way ANOVA and Bonferroni as post-hoc test).

Al exposure to the low dose of 8.3 mg/kg for 60 days (Group 1b) and to the higher dose of 100 mg/kg for 42 days (Group 2b) increased the number of activated microglia in hippocampus (Fig. 6B,E), being 3.4 and 4.5 times higher, when compared with the respective control groups (Fig. 6G: Al 8.3 vs Ct P = 0.0053; Fig. 6H: Al 100 vs Ct P = 0.0023). Moreover, the overall score for COX-2 immunoreactivity in the hippocampal tissue was 3.4 in Al-treated rats at Group 1 and 3.8 for Group 2, significantly higher when compared with the control groups (2.0 and 2.2, for Group 1a and Group 2a, respectively) (Fig. 7B,E,G: Al 8.3 vs Ct P = 0.0261; 7 H: Al 100 vs Ct P = 0.0018). Al-treated rats and co-treated with EWH (Group 1d and Group 2d) showed number of activated microglia similar to control-rats (Fig. 6C,F,G: Al 8.3 + EWH vs Al P = 0.0201; 6 H: Al 100 + EWH vs Al P = 0.0302) and weakly positive immunoreactivity for COX-2 in the group 1d, treated with Al at the lowest dose (Fig. 7C,F,G: Al 8.3 + EWH vs Al P = 0.0201; 7 H: Al 100 + EWH vs Al P = 0.634).

### Presence of aluminum in the hippocampus

**Figure 8:**
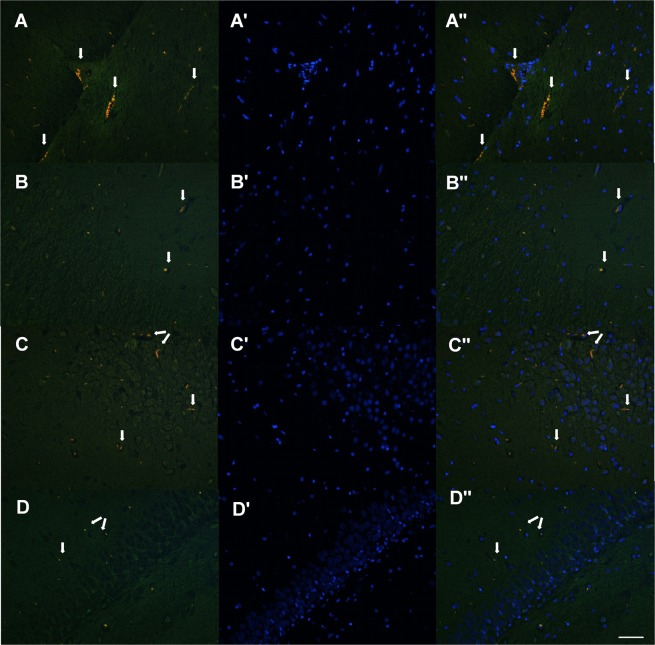
Effect of EWH on the presence of aluminum (orange) in hippocampus. Representative images of Al in the hippocampus: lumogallion fluorescence in rats treated with AlCl_3_ at 8.3 mg/kg (**A**) and co-treated with EWH (**B**), lumogallion fluorescence in rats treated with AlCl_3_ at 100 mg/kg (**C**) and co-treated with EWH (**D**). DAPI-staining (blue), (A’–D’); lumogallion & DAPI overlay (A”–D”). Arrows indicate the specific presence of aluminum. Scale bar: 50 µm (objective X20).

The presence of Al in the hippocampus was confirmed using lumogallion and fluorescence microscopy. The tissue showed green autofluorescence in the absence of lumogallion (supplementary information, Fig. SIA) and no specific fluorescence in the control (Group 1a and Group 2a) and EWH rats (Group 1c and Group 2c) (supplementary information, Fig. SIB & SIC). Lumogallion fluorescence identified Al in the hippocampus of Al-treated rats as evidenced by bright orange fluorescence (Fig. 8A,C), using lumogallion fluorescence, DAPI- fluorescence and lumogallion & DAPI overlay. Rats in the Al + EWH groups (Group 1d and Group 2d) showed weakly orange fluorescence (Fig. 8B,D).

## Discussion

The results of our study show that Al exposure at both normal and high human dietary level impairs short and long term memory and promotes catalepsy. Moreover, our results point out that in addition to oxidative stress and cholinergic dysfunction, inflammation though microglia and COX-2 activation is a way by which Al can induce toxicity in the hippocampus. The present study has investigated the effects of dietary supplementation with EWH, with known antioxidant and anti-inflammatory properties, against Al effects. Our findings suggest that the EWH could be used as a functional food ingredient to prevent or minimize Al effects after long-term exposure. Namely, EWH prevents cognitive impairments, cholinergic dysfunction and hippocampal oxidative stress and inflammation induced by Al. The current study shows the specific presence of Al in the hippocampus of Al-exposed rats, however further detailed study will be necessary to verify the precise locations of deposits of Al in the brain.

Al is neurotoxic and its toxicity has been demonstrated as the main cause of encephalopathy in renal dialysis patients^46^. Over the past few decades the so-called “aluminum hypothesis” on AD has been debated and investigated through experimental and clinical studies and, now AD is considered as an acute response to chronic intoxication by Al^8,47^. Al toxic effects appear when the Al content of any tissue achieves a toxic threshold or burden^47^. Recently, we have demonstrated that Al exposure at human dietary level reaches a threshold sufficient to promote cognitive dysfunction^30^.

Environmental accidents can expose people to high concentrations of Al^48,49^. One such accident occurred on July 6th 1988 in Camelford, Cornwall, UK when 20 tons of aluminum sulphate was discharged into the mains water supply. Up to 20 000 people were exposed to concentrations of Al which were 500–3000 times the acceptable limit under European Union legislation (0.200 mg/L)^48^. Since that, three follow-up studies were carried out reporting severe cognitive decline, unspecified neuropathological features and high brain Al levels from two individuals who were living in the Camelford area at that time^44,50,51^. The general population is exposed to Al every minute and, due to myriad Al applications and sources, most of them are exposed to Al at a level that exceeds the tolerable weekly intake recommended by International Regulatory Agencies^4–7^. Therefore, strategies worldwide that aim to reduce human Al exposure or prevent the effects of this burgeoning and silent poison are needed. The current study shows that a functional food ingredient which could be easily introduced in the human diet can minimize the cognitive effects of Al exposure by diet.

But how does EWH exerts its effects? EWH is produced after 8 h of hydrolysis digestion with pepsin releasing bioactive peptides with *in vitro* peroxyl radical-scavenging activity (574 μmol Trolox/g protein). Among the identified peptides, amino acid sequences with Pro, Lys or Arg as a C-terminal residue exhibit ACE inhibitory activity, Arg or Tyr at the N-terminal position show vascular-relaxing activity and, finally the presence of Tyr and Phe in C-terminal residue is related to scavenging free radicals and antioxidant properties^19^. *In vivo*, the EWH improves obesity-related complications^20,52^, cardiovascular^53^, reproductive^22^ and neurologic disorders after chronic exposure to metals^24^ and, these effects were related to its antioxidant and anti-inflammatory capacities. In the present study, rats treated with Al and co-treated with EWH did not develop memory and behavior dysfunctions after Al exposure, suggesting the efficacy of EWH to counteract the adverse effects after Al exposure at low and high levels.

Al is a neurotoxin in which effects could be maximized in individuals that share susceptibility factors, such as environmental or genetic traits^9^. Al^3+^ is a potent pro-oxidant, its toxicity being strongly related to oxidative stress in several organs^12,14,17^. Furthermore, experimental animals exposed to the adjuvant Al oxyhydroxide (Alhydrogel^®^) or to AlCl_3_ at low doses showed activation of the innate immune system with increased numbers of macrophage and microglia together with high Al levels^12,14,54^. Here we found that besides oxidative stress and cholinergic dysfunction, the presence of Al in the hippocampus promoted microglial activation, which in turn appears to induce the inflammatory COX-2 cascade activation. In agreement, we have recently seen that ROS and COX-2-derived prostanoids are important mediators of vascular dysfunction after Al exposure^12^.

In the present study, we demonstrated the capacity of the EWH to counteract the behavioral dysfunction induced by Al. Namely, the co-treatment with EWH prevents memory impairment and catalepsy development after Al exposure. Among the mechanisms of actions, ours results suggest that the EWH prevents the inhibition of cholinergic activity and increased oxidative stress and inflammation. However, there are numerous ways by which EWH could be acting to counteract the Al effects, for example acting on the gastrointestinal tract. Besides the multiple functionalities of the GI tract, such as absorption of dietary components and immune regulation, the gastrointestinal tract is the main and first route of contact with the ingested Al, influencing its bioavailability and absorption^55^. Therefore, it is possible to speculate that EWH effects could originate in this important target organ for Al deposition. Moreover, the ameliorative effects of the EWH on cardiometabolic complications in *Zucker* obese rats were related to its action on the gastrointestinal tract^21^. However, at this moment we are still not able to affirm which mechanism (s) is/are responsible for the beneficial effect of the EWH. We should consider that both, Al and EWH, are being administered through oral routes which may appoint for two main protective mechanisms: 1). EWH can reduce/inhibit the absorption of Al across the GI tract, then reducing the effects of Al into the body or, 2). EWH, maybe by its antioxidant and anti-inflammatory properties, can inhibit/prevent the consequences of the enhanced human body burden of Al.

## Conclusion

Taken as a whole, our data suggest that the EHW could be used as a supplementary dietary ingredient to provide a strategy against Al toxicity. Our results show that daily intake of Al at a concentration achieved through the human diet impairs short and long term memory and induces catalepsy, effects similar to those found after Al exposure at a high level. Moreover, besides oxidative stress and cholinergic dysfunction, our results highlight inflammation though microglia and COX-2 as a way by which Al can induce toxicity in the hippocampus. The co-treatment with EWH is able to partially prevent the presence and effects of Al in the brain. Therefore, EWH could be used as a functional food ingredient to protect against the toxicity of aluminum in the human diet.

## Supplementary information

Supplementary Figure S1
