# Supplementary material for: Egg White Hydrolysate as a functional food ingredient to prevent cognitive dysfunction in rats following long-term exposure to aluminum
Source: Sci Rep. 2019 Feb 12;9:1868. doi: 10.1038/s41598-018-38226-7 (PMC6372713; doi:10.1038/s41598-018-38226-7)

Supplementary Information

Egg White Hydrolysate as a functional food ingredient to prevent cognitive dysfunction in rats following long-term exposure to aluminum

Caroline Silveira Martinez[a**], Caroline D. C. Alterman[a], Gema Vera[b], Antonio Márquez Gallego[b], José Antonio Uranga Ocio[b], Franck Maciel Peçanha[a], Dalton Valentim Vassallo[c], Christopher Exley[d], Pâmela B. Mello-Carpes[a], Marta Miguel[e*], Giulia Alessandra Wiggers[a]

Figure legend

Fig SI. Effect of EWH on the presence of aluminum in hippocampus. Autofluorescence (green) (A), lumogallion fluorescence (orange) for Al in control (B) and EWH-treated animals (C) showing no specific orange fluorescence. DAPI-staining (blue), (A'), (B') & (C'); autofluorescence or lumogallion & DAPI overlay (A''), (B'') & (C''). Scale bars: 50 µm (objective X20).

Figure SI

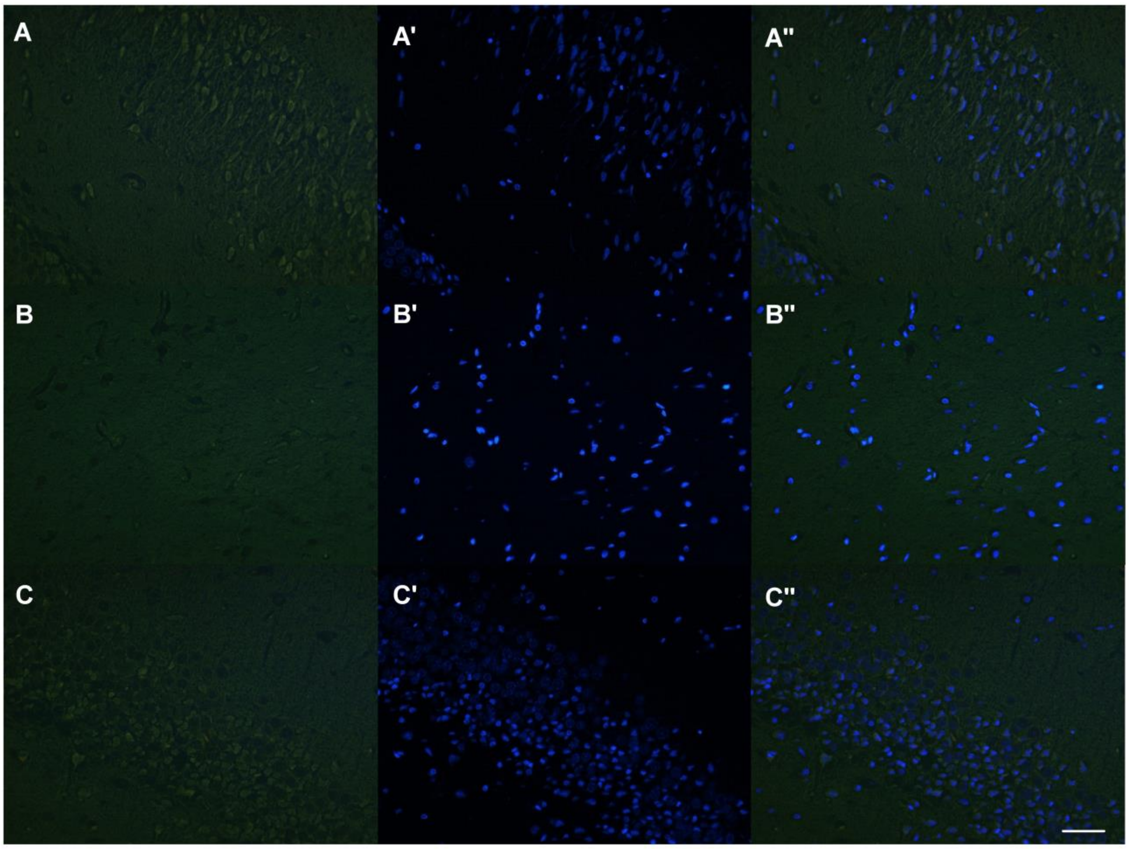

Supplement: Supplementary file 1 — Supplementary Figure S1 [file 41598_2018_38226_MOESM1_ESM.pdf]
